# Virtual Screening for SARS-CoV-2 Main Protease Inhibitory Peptides from the Putative Hydrolyzed Peptidome of Rice Bran

**DOI:** 10.3390/antibiotics11101318

**Published:** 2022-09-27

**Authors:** Nathaphat Harnkit, Thanakamol Khongsonthi, Noprada Masuwan, Pornpinit Prasartkul, Tipanart Noikaew, Pramote Chumnanpuen

**Affiliations:** 1 Medicinal Plant Research Institute, Department of Medical Sciences, Ministry of Public Health, Nonthaburi 11000, Thailand; 2 Mahidol Wittayanusorn School, 364 Salaya, Phuttamonthon District, Nakhon Prathom 73170, Thailand; 3 Department of Biology and Health Science, Mahidol Wittayanusorn School, 364 Salaya, Phuttamonthon District, Nakhon Prathom 73170, Thailand; 4 Omics Center for Agriculture, Bioresources, Food and Health, Kasetsart University (OmiKU), Bangkok 10900, Thailand; 5 Department of Zoology, Faculty of Science, Kasetsart University, Bangkok 10900, Thailand

**Keywords:** antiviral peptide, bioinformatics, rice bran, SARS-CoV-2 main protease, molecular docking, molecular dynamics

## Abstract

The Coronavirus Disease 2019 (COVID-19) caused by the severe acute respiratory syndrome coronavirus 2 (SARS-CoV-2) has led to the loss of life and has affected the life quality, economy, and lifestyle. The SARS-CoV-2 main protease (Mpro), which hydrolyzes the polyprotein, is an interesting antiviral target to inhibit the spreading mechanism of COVID-19. Through predictive digestion, the peptidomes of the four major proteins in rice bran, albumin, glutelin, globulin, and prolamin, with three protease enzymes (pepsin, trypsin, and chymotrypsin), the putative hydrolyzed peptidome was established and used as the input dataset. Then, the prediction of the antiviral peptides (AVPs) was performed by online bioinformatics tools, i.e., AVPpred, Meta-iAVP, AMPfun, and ENNAVIA programs. The amino acid composition and cytotoxicity of candidate AVPs were analyzed by COPid and ToxinPred, respectively. The ten top-ranked antiviral peptides were selected and docked to the SARS-CoV-2 main protease using GalaxyPepDock. Only the top docking scored candidate (AVP4) was further analyzed by molecular dynamics simulation for one nanosecond. According to the bioinformatic analysis results, the candidate SARS-CoV-2 main protease inhibitory peptides were 7–33 amino acid residues and formed hydrogen bonds at Thr22–24, Glu154, and Thr178 in domain 2 with short bonding distances. In addition, these top-ten candidate bioactive peptides contain hydrophilic amino acid residues and have a positive net charge. We hope that this study will provide a potential starting point for peptide-based therapeutic agents against COVID-19.

## 1. Introduction

The COVID-19 pandemic leads to a high mortality rate, affects the work system, food system, and economy, and causes changes in human lifestyles [1]. Moreover, infection with Severe Acute Respiratory Syndrome Coronavirus-2 (SARS-CoV-2) provokes a mutation of COVID-19 or Coronavirus Disease 2019 [1,2]. The virus passes through the body into the respiratory tract, where angiotensin-converting enzyme 2 (ACE2), which is present on the cell surface, binds to the viral spike protein and enables the virus to enter the cell. Since ACE2 can capture the virus on it spike protein, the virus can enter the host cell. The main protease (Mpro) of SARS-CoV-2 plays a pivotal role in mediating the replication and transcription of viral genes. Mpro hydrolyzes the polyprotein at at least eleven conserved sites and begins by cleaving the pp1a and pp1b of Mpro. For this reason, finding Mpro inhibitors can help reduce the spread of COVID-19. As a result, proteases are attractive antiviral targets [1]. One of the consequences of the COVID-19 epidemic is the shortage of resources and materials for drug research and development. Natural extracts represent an attractive option due to the ready availability of materials.

Since plant seeds naturally accumulate proteins for long-term storage, they are suitable for the production of stable and large amounts of antimicrobial peptides. In particular, rice seed and rice bran are considered good protein sources [3]. In rice (*Oryza sativa*), 60–80% of all storage proteins are glutelins, 20–30% are prolamines, and about 5% are globulins [4,5,6]. Interestingly, there are several reports on the broad biological activities of proteins and peptides derived from rice bran, i.e., anti-cancer, anti-inflammatory, anti-diabetic, therapy for chronic diseases, and anti-COVID-19 effects [7,8,9,10,11,12].

Bioinformatics is the application of tools of computation and analysis to capture, interpret, and evaluate data in molecular biology. A great benefit of using bioinformatics is that it can save time and resources. Moreover, it provides a base for creating three-dimensional models of complex molecules [13]. Furthermore, it is safer in terms of cost and time to use bioinformatics in research than in the laboratory. Recently, docking studies of some rice bran peptides with the integrin αIIbβ3 and ACE 2 receptors demonstrated that the rice peptide has potential against SARS-CoV-2 infection [12]. However, the antiviral peptide screening of the putative hydrolyzed rice bran peptidome and molecular docking of the SARS-CoV-2 main protease have not been studied.

Therefore, this study aimed to develop a bioinformatic workflow for the virtual screening of antiviral peptides and use in silico protein–peptide docking studies to select the inhibitory peptide for the SARS-CoV-2 main protease.

## 2. Results and Discussion

### 2.1. Rice Bran Putative Antiviral Peptide Screening using a Computational Method

**Figure 1:**
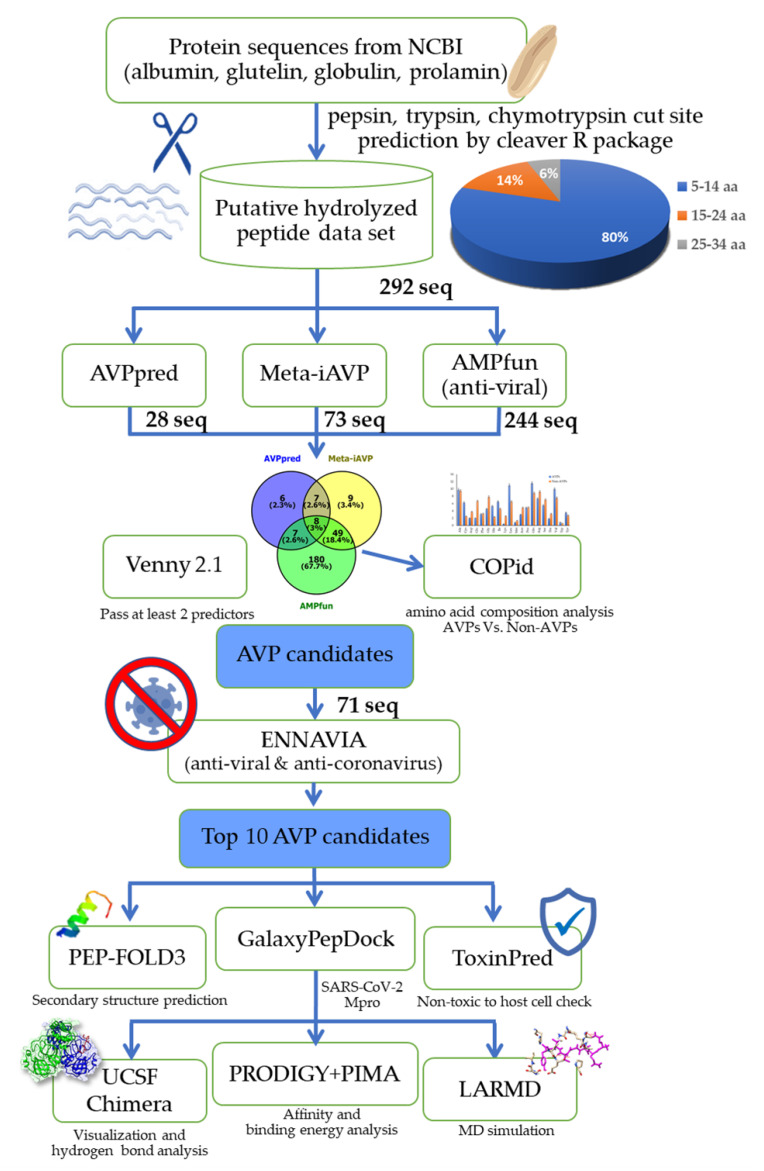
The workflow of the bioinformatic virtual screening for antiviral peptide (AVP) candidates and the in silico analysis of the SARS-CoV-2 main protease inhibition using molecular docking simulation.

**Figure 2:**
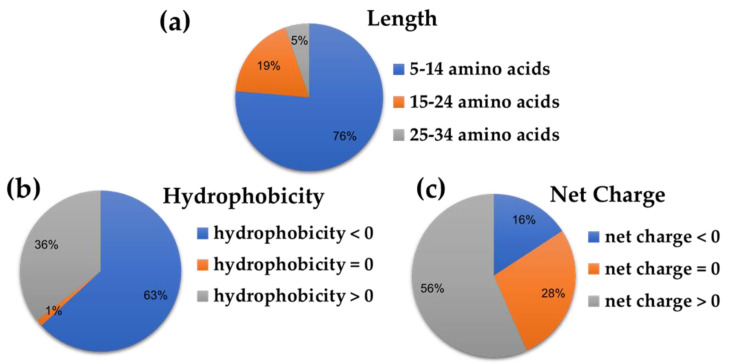
Percentage of the peptides’ properties with respect to their (**a**) length, (**b**) hydrophobicity, and (**c**) net charge based on 71 putative AVPs.

All of the peptide sequences from this putative peptidome of rice bran (*Oryza sativa*) are shown in the supplementary file (Table S1) as the predicted cut results of specific protease enzyme and original protein sources. The peptides were predicted to be antiviral peptides (AVPs) if the cut-off criteria at the support vector machine (SVM) probability exceeded 50 in AVPpred (either in Model 3: Composition or Model 4: Physicochemical properties) and the probability was greater than 0.5 in Meta-iAVP and AMPfun (only antiviral activity). As a result, AVPpred predicted 28 AVPs; Meta-iAVP predicted 73 AVPs; AMPfun predicted 244 AVPs; and there are 71 AVPs in common from at least two prediction servers from 292 input peptide sequences (Figure 1). In terms of the peptides’ properties (Figure 1), the majority of putative AVPs were 5–14 amino acids long (76%), hydrophilic (63%), and cationic (56%) (Figure 2).

Focusing on the length distribution of candidate AVPs and upon further analysis of the peptide length determining antiviral activity, it was observed that 58 (76%) and 14 (19%) were 5–14 and 15–24 amino acids in length, respectively. In general, AVPs are usually short (8–40 amino acids in length) and consist of cationic amino acid residues [14,15,16,17,18,19]. The most optimal peptide length for AMPs, especially AVPs, is 10–40 amino acid residues and hence, it is of great value to optimize the AVP length [20,21].

**Figure 3:**
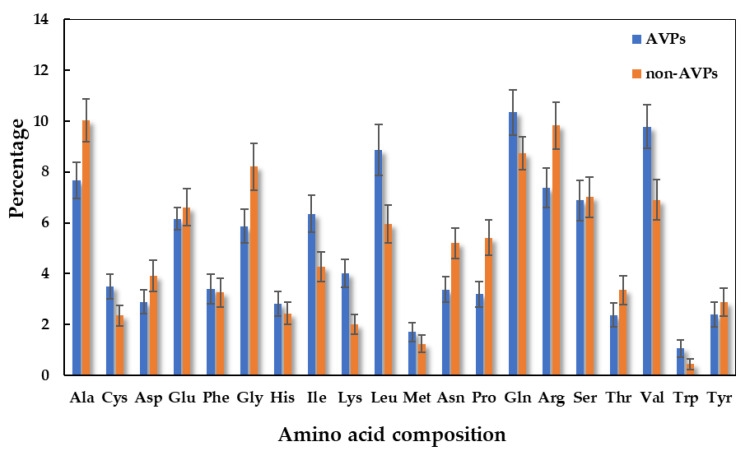
Compositional analysis represents the preferences between the significant AVPs and non-AVPs.

A comparison of the amino acid composition of AVPs is shown in Figure 3. Compared to the non-AVPs, the preferential amino acid residues with higher percentages were Lys, Leu, Ile, Val, Trp, and Gln. This is consistent with what have been reported before for AVPs, which work as antimicrobial peptides and possess cationic and amphipathic characteristics with positive net charges [14,22,23]. In particular, this specific basic residue (Lys) is the preferential amino acid for antiviral activity and is abundantly found as a component of therapeutic peptides with enhanced electrostatic properties [14]. This also facilitates the interaction and insertion of peptides into the anionic cell walls and phospholipid membranes of microorganisms [22,24]. Therefore, Lys (not Arg or His) plays an important role in providing cationic properties to enhance the antiviral activities of AVPs [14,25]. However, the reason for AVPs bias toward lysine is not clear: either their potential to inhibit the viral enzymes or the inhibition of the viral-entering process [14,26]. On the other hand, the medium-sized hydrophobic residues (Leu and Ile) were also found to be the most abundant residues in the AVPs and play important role in the amphipathic characteristics of antimicrobial peptides [14,27].

### 2.2. Prediction Scores and Physicochemical Properties of the Ten Top-ranked AVPs

The selection of the ten top-ranked AVPs was based on their predictive probability scores or SVM scores as mentioned in the previous section. The predictive scores and amino acid sequence details of the ten top-ranked antiviral peptides predicted from the putative rice bran peptidome by using four bioinformatics programs are shown in Table 1. Since the secondary structure is one of the most important peptide sequence features for predicting AVPs, each peptide secondary structure was predicted by PEP-FOLD3. These peptides were structurally classified as two main groups; random coiled (AVP1, 2, 4, 9, and 10) and helix-containing loops (AVP3, 5, 6, 7, and 8). Consistent with previous studies, random coils and α-helices were reported to be the top two dominant secondary structures of AVPs compared to the β-sheet structure [14,28]. 

All of the predictive scores that were greater than the cut-off criteria (the support vector machine (SVM) probability exceeded 50 in AVPpred and the probability was higher than 0.5 in Meta-iAVP and AMPfun) were highlighted in red. Even though there are four SVM-based models available in the AVPpred program based on the peptide sequence features, only the amino acid compositions (Model 3) and physiochemical property (Model 4) models can provide predictive scores. Since the antiviral peptide motifs (Model 1) and sequence alignment (Model 2) could give only two classifications (AVPs and Non-AVPs), we only took the predictive SVM scores from Model 3 (M3) and Model 4 (M4) for this particular AVP candidate ranking. In the ENNAVIA program, there are two main sets of the neural network prediction models. The first two models were used for antiviral property classification (ENNAVIA-A and B, based on the antiviral vs. non-antiviral datasets and antiviral vs. random datasets, respectively) while the other two models were used for anti-coronavirus property prediction (ENNAVIA-C and D, based on the anti-coronavirus vs. non-antiviral datasets and anti-coronavirus vs. random datasets, respectively) [29]. 

The calculated scores of the physicochemical properties of all of the selected AVP candidates (AVP1 to AVP10) obtained from the ToxinPred web server are shown in Table 2. The largest peptide was AVP4 with a molecular weight of 3916.01 g/mole while AVP10 was the smallest peptide with a molecular weight of 792.01 g/mole. All top-ranked putative AVPs were classified as amphipathic characteristics with steric hindrance and sidebulk scores higher than 0.5. 

### 2.3. Protein–peptide Docking Simulations

**Figure 4:**
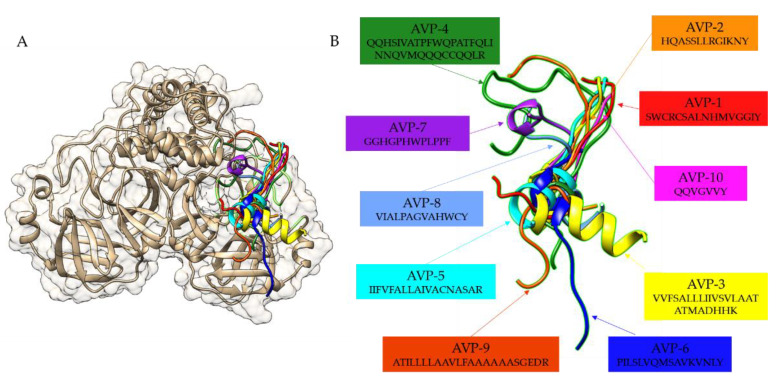
Comparative molecular docking of the ten top-ranked AVPs (AVP1 to AVP10) on the crystal structure of the SARS-CoV-2 main protease in the apo state (PDB ID: 7C2Q) shown with (**A**) and without (**B**) the enzyme structure. The structure of the SARS-CoV-2 main protease is shaded in gold, and the peptides are labeled with different colors.

**Figure 5:**
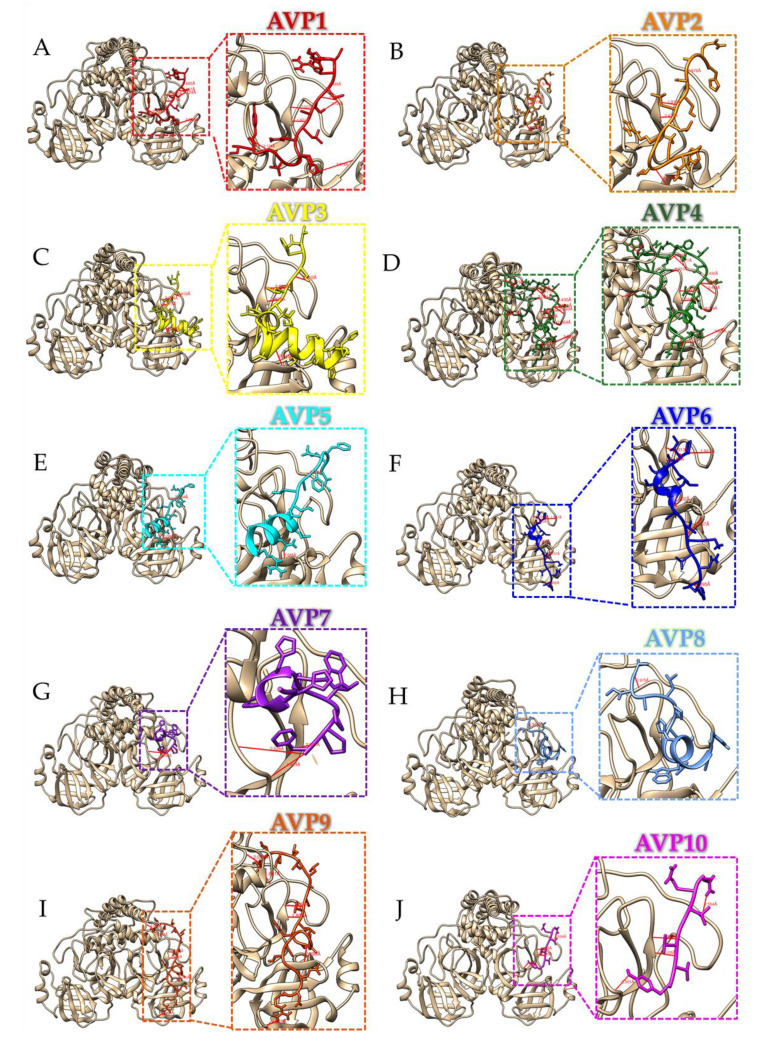
Molecular docking of the ten top-ranked AVPs (AVP1 (**A**), AVP2 (**B**), AVP3 (**C**), AVP4 (**D**), AVP5 (**E**), AVP6 (**F**), AVP7 (**G**), AVP8 (**H**), AVP9 (**I**), and AVP10 (**J**)) to the crystal structure of the SARS-CoV-2 main protease in the apo state (PDB ID: 7C2Q). The structure of the SARS-CoV-2 main protease is shaded in gold, and the peptide sequences are colored as labeled above. The hydrogen bonds are shown as red lines.

Bioinformatics prediction of the SARS-CoV-2 main protease inhibitory peptides is challenging due to lack of the computational predictors available. The molecular docking of these ten top-ranked AVPs to the crystal structure of the SARS-CoV-2 main protease demonstrated that AVP1 to AVP10 binds near the active site of the SARS-CoV-2 main protease structure (Figure 4). Based on the visualization of the protein–peptide docking simulation results, all hydrogen bonds observed from the molecular docking of ten peptides (AVP1 to AVP10) to the crystal structure of the SARS-CoV-2 main protease are listed in Table 3. In general, the proper hydrogen bond acceptor–donor pair is within the correct distance (2.7 to 3.3 Å) [30], and most hydrogen bonds between peptide molecules and protein macromolecules are relatively strong, starting with a 3 Å bond length [31]. According to our molecular docking results, the distance of the hydrogen bonds between selected AVPs and the SARS-CoV-2 main protease structure ranged from 1.8 to 3.8 Å. In particular, hydrogen bonds with donor–acceptor distances of 2.2–2.5 Å are considered to be “strong, mostly covalent” while those of 2.5–3.2 Å and 3.2–4.0 Å are considered to be “moderate, mostly electrostatic” and “weak, electrostatic”, respectively [32]. A comparison of the interaction of the SARS-CoV-2 main protease structure indicated that all selected AVPs showed similar binding positions to the SARS-CoV-2 main protease structure (Figure 4). All ten selected AVPs could interact with the amino acid residues near the active site of the SARS-CoV-2 main protease by hydrogen bonding (Figure 5 and Table 3). The top-ranked AVP candidate (AVP1) with the highest predictive anti-coronavirus activity score formed five hydrogen bonds (with Gln180, Thr178, Glu154, Cys42, and Gly131). Notably, the largest AVP candidate (AVP4) interacted with 19 residues by forming 24 hydrogen bonds near the active site area, mainly the Gln22 and Arg33 residues (Figure 5 and Table 3). 

The docking positions of all selected AVPs closer to the active site of the SARS-CoV-2 main protease structure were very similar to the binding pocket of the known main protease inhibitory marine polyketides [33] and antiviral drugs [34]. The inhibitory peptides tended to form hydrogen bonds with the Thr22–24, Glu154, and Thr178 residues of the SARS-CoV-2 main protease. These hydrogen bonds were found in very short distances (1.8–2.5 Å) and are considered to be “strong, mostly covalent” interactions, indicating a high binding affinity. 

According to the binding affinity and binding energy analysis by the PROGIDY and PIMA web servers (Table 4), AVP4 showed the strongest binding to the binding groove of the SARS-CoV-2 main protease enzyme with a molecular docking score of −363.04 kJ/mol and a binding affinity (ΔG) of −14.5 (kcal/mol). This high docking score was better than the docking scores of SARS-CoV-2 Mpro for several drugs, i.e., noscapine, chloroquine, ribavirin, and favipiravir (−292.42, −269.71, −214.17, and −153.91 kJ/mol, respectively) [34]. The Mpro–AVP4 interface interactions involved in affinity and binding energy distribution were obtained by high van der Waals energy, −325.48 kcal/mol (hydrophobic interactions) and hydrogen bond energy −37.56 kcal/mol, which are considered to be the most significant calculations to assess the binding stability. Based on the molecular docking results of this study, it was proven that the selected AVPs have potential as candidates for the SARS-CoV-2 main protease inhibitor in controlling the COVID-19 disease.

### 2.4. Simulation of the Molecular Dynamics of the Mpro–AVP4 Complex 

**Figure 6:**
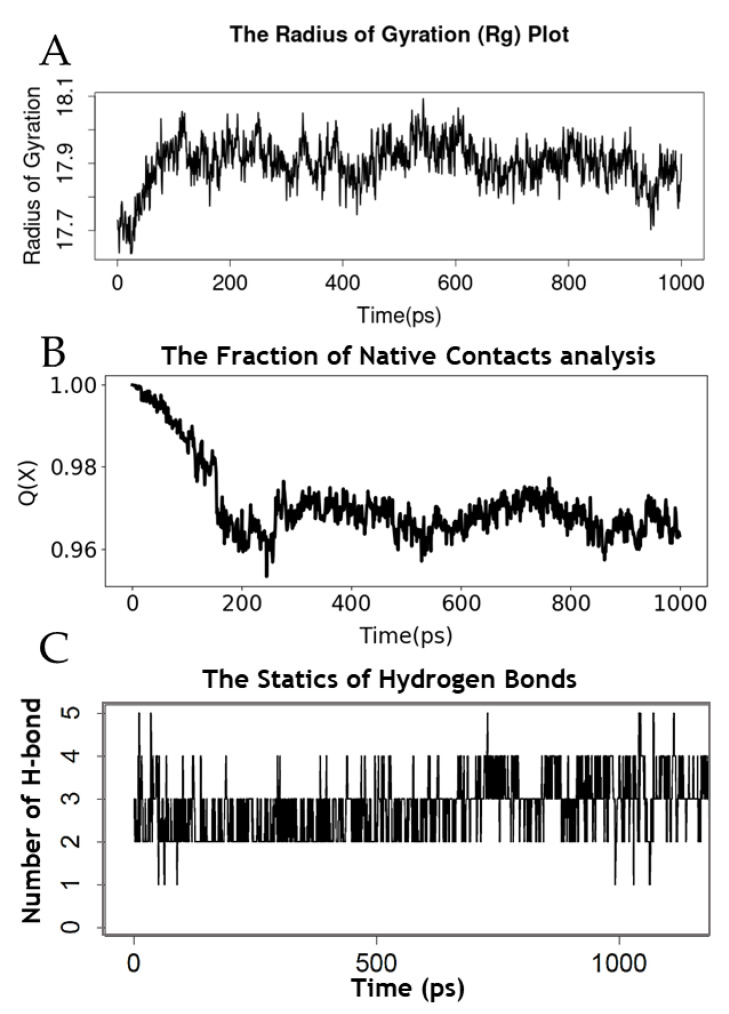
Molecular dynamics simulation of the Mpro–AVP4 complex; radius of teg gyrus plot (**A**), fraction of native contact analysis (**B**), and statistics of the hydrogen bonds (**C**).

Based on the highest ranked binding affinity and docking scores, the AVP4 binding to the Mpro enzyme was assessed by molecular dynamics simulation for one nanosecond to examine the conformational stability and fluctuation analysis of the complex. To determine the structural activity of the macromolecule, the radious of gyration (Rg) of resulting trajectories was calculated. The Rg level varied according to the folding state of the protein–peptide complex and fluctuated between 17.7 and 18.0 Å, and minimal fluctuations showed the stability of the Mpro macromolecule while binding to AVP4 (Figure 6A). After that, the fraction of the native contacts Q of trajectories was analyzed to define the transition states for protein folding (conformational changes) with free energy. The result depicted that the native contacts were favored by the coarse-grained theoretical models. Q values were determined to be above 96% and depicted the conformational dynamics of the Mpro enzyme along with the energetics of bound ligands (AVP4) (Figure 6B). Moreover, the hydrogen bonds were analyzed to reveal the dynamic equilibration of the complex system with a high number of hydrogen bonds and demonstrated the stable binding of AVP4 with the target Mpro enzyme (Figure 6C). These analyses demonstrated the prolonged and robust binding of AVP4 with target Mpro of coronavirus and the involvement of the potential binding energies with the correlation of the MD calculations and the stability of the Mpro enzyme−AVP4 complex.

### 2.5. In Silico Toxicity Analysis of the Selected AVPs

Toxic side effects are usually considered to ensure high specificity and low cross-reactivity when designing effective, safe, and theoretically infallible therapeutic molecules. In particular, the computational screening of non-toxic peptide approaches is required to improve the selectivity of therapeutic peptides with less cost and time [35]. The online bioinformatic tool, ToxinPred, was used to predict and estimate the toxicity of the putative AVPs to the host cell. There are eight predictive models available for peptide toxicity analysis; SVM (Swiss-Prot) based, SVM (Swiss-Prot) + Motif based, SVM (TrEMBL) based, SVM (TrEMBL) + Motif based, Monopeptide (Swiss-Prot), Monopeptide (TrEMBL), Di-peptide (Swiss-Prot), and Dipeptide (TrEMBL). In order to comprehensively profile the potential toxic side effects to the host cells, all eight models were used for this particular analysis. All of the predictive scores can be observed in Table 5. The negative scores indicate non-toxic classified results while the positive scores indicate the possible toxicity of the analyzed peptides (highlighted in red). Three selected AVPs (AVP1, 7, and 8) were predicted to be potentially toxic in some predictive models (only from the Quantitative Matrix (QM) method). The rest of the candidates (AVP2 – 6, 9, and 10) had ToxinPred scores lower than the others, suggesting less possible toxicity to the host cells.

Based on the fact that the ToxinPred server can predict the toxicity or non-toxicity of peptides with higher accuracy in the SVM method (93.92%) compared to the QM method (88.00%) [36], the SVM model seems to be more reliable. Since there are some other factors involved in the real biological systems (i.e., secondary structure, in vivo instability, the bioactive activity at a specific pH, temperature, and tonicity, etc.), laboratory experiments would still be required to confirm the real toxicity of all candidate peptides.

Even though the therapeutic peptides have a broad spectrum of targets and low toxicity in general, there are also some limitations and challenges of therapeutic peptide development to be considered for further applications [37]. For example, most of them are limited in oral bioavailability with a short half-life and rapid clearance, and some of them contain immunogenic sequences with some potential to cause an allergenic effect in some patients [37,38]. To overcome these challenging problems, several optimized solutions have been proposed by researchers. A previous study suggested that the side chain of non-polar aromatic amino acids (Trp and Phe) can promote peptide structural stability by restricting their conformation through hydrogen bond formation [39]. In addition, it has been reported that non-polar aliphatic amino acids (Ala, Ile, Leu, and Val) are responsible for the thermal stability of proteins and peptides [40]. Taking this constraint as a guideline, we could selectively screen and/or redesign peptides to improve not only the stability of the therapeutic peptides but also other physicochemical properties involving specific cellular and molecular functions. Beside ToxinPred, there are also some web servers for allergenic peptide prediction, i.e., AllerTOP [36] and AllerFP [41], to avoid possible side effects of therapeutic peptides on the host cells [42,43]. 

## 3. Materials and Methods

According to the pipeline illustrated in Figure 1, we proposed the bioinformatic virtual screening workflow with in silico validation by protein–peptide molecular docking. The workflow begins with the predicted digestion peptidomes of the four major proteins in rice bran; albumin, glutelin, globulin, prolamin, with three protease enzymes (pepsin, trypsin, and chymotrypsin). The reason for this putative peptidome of rice bran hydrolyzed by these three proteases was that there are several relevant research reports on the high potential bioactivities of enzymes prepared rice bran protein hydrolysates using digestive enzymes [44]. The functional peptides from rice bran prepared by pepsin, trypsin, and chymotrypsin showed high efficiency and antioxidant bioactivity [45], as well as ACE-inhibitory [46], antimicrobial [47], and tyrosinase-inhibition activity [48]. Having specific cut sites on polypeptide sequences, pepsin, trypsin, and chymotrypsin have been beneficially used for amphipathic and/or cationic therapeutic peptide screening from food-derived peptides [44]. In this study, the putative hydrolyzed peptidome was established and used as the input datasets of 292 peptides for the selection of the peptides with the ten top-ranked predicted scores of unique AVPs (also predicted as non-toxic to the host cells).

### 3.1. Preparation of the Rice Bran Putative Hydrolyzed Peptidome

Since several predictive programs (PEP-FOLD 3.5, ENNAVIA, and GalaxyPepDock) require a proper length for their machine learning-based analyses, only those peptides that consisted of at least 6 amino acid residues were further screened. After the removal of short peptides (fewer than 5 amino acids), 292 rice bran peptide sequences were obtained from the predicted cut sites of the four major proteins of rice bran (*Oryza sativa*: Uniprot taxonomic ID = 4530); albumin, glutelin, globulin, prolamin (from the National Center for Biotechnology Information: NCBI with specific accession numbers; Q01882, Q40689, O65042, and Q40714, respectively). The in silico pepsin, trypsin, and chymotrypsin digestions were performed by the cleaver R package (version: 1.34.1; [49]). There were 3 groups of peptide length distributions, i.e., 5–14 amino acid residues (233 sequences, 80%), 15–24 amino acid residues (42 sequences, 14%), and 25–34 amino acid residues (17 sequences, 6%) (Figure 1).

### 3.2. The Bioinformatic Prediction and Screening of Antiviral Peptides

The peptide sequences were arranged in FASTA format and were used as input to predict the antiviral properties using 3 online machine learning-based prediction programs, i.e., AVPpred (http://crdd.osdd.net/servers/avppred/submit.php, accessed on 10 January 2022) [50], Meta-iAVP (http://codes.bio/meta-iavp/, accessed on 10 January 2022) [22], and AMPfun (http://fdblab.csie.ncu.edu.tw/AMPfun/run.htm, accessed on 10 January 2022l) [51]. Since there are several bioactivity models to choose from in AMPfun, only the antiviral classification model was considered in this particular study. Venny 2.1.0. (https://bioinfogp.cnb.csic.es/tools/venny, accessed on 12 January 2022) [52] was used to generate Venn diagrams to visualize the significant positive AVP candidates that were classified as AVPs at least by 2 programs from 3 online bioinformatic prediction tools. To obtain the probability score of anti-coronavirus activity, all significant AVP candidates were analyzed with the neural network peptide antiviral and anti-coronavirus activity predictor ENNAVIA (https://research.timmons.eu/ennavia, accessed on 13 January 2022) [29]. The average scores from 4 predictive models of ENNAVIA (2 antiviral and 2 anti-coronavirus predictive models) together with 3 other AVP predictors were considered to rank all of the AVP candidates. Only the ten top-ranked AVPs were selected for further molecular docking performances regarding the SARS-CoV-2 main protease inhibition. To predict the secondary structure of the selected peptides, PEP-FOLD3.0 (https://bioserv.rpbs.univ-paris-diderot.fr/services/PEP-FOLD3, accessed on 15 January 2022) was used to simulate the feasible molecular structure of the ten top-ranked antiviral peptides. Finally, all 8 predictive models based on SVM methods and the Quantitative Matrix (QM) method in ToxinPred (https://webs.iiitd.edu.in/raghava/toxinpred/protein.php, accessed on 15 January 2022) were used to predict whether the peptides were cytotoxic to the host cells. All calculated scores of the physicochemical properties of the selected AVP candidates were also obtained by the ToxinPred web server in the batch submission option.

### 3.3. The Protein–peptide Molecular Docking Simulation 

The crystal structure of the SARS-CoV-2 main protease in the apo state with PDB ID code 7C2Q [53] was accessed from the Protein Data Bank (PDB) (http://www.rcsb.org, accessed on 10 January 2022). 

The amino acid sequences of each selected AVP candidate (AVP1 to AVP10) were docked to the SARS-CoV-2 main protease enzyme using the GalaxyPepDock (http://galaxy.seoklab.org/pepdock, accessed on 21 January 2022)) [53]. The docking results of the best model and hydrogen bond finding were visualized by the UCSF Chimera program [54]. The FindHBond tool (in the menu under Tools and Structure Analysis) was used to identify and analyze the H-bonding patterns between each AVP candidate and SARS-CoV-2 main protease. To investigate the protein–peptide interface interactions involved in affinity and binding energy, PIMA [55], available at http://caps.ncbs.res.in/pima (accessed on 24 January 2022), and PRODIGY [56] (https://wenmr.science.uu.nl/prodigy, accessed on 24 January 2022) web servers were employed.

### 3.4. Molecular Dynamics Simulation Analysis of AVP with the Mpro Enzyme

The molecular dynamics of the Mpro–AVP docked complexes were determined using the Ligand and Receptor Molecular Dynamics (LARMD) web server (http://chemyang.ccnu.edu.cn/ccb/server/LARMD/, accessed on 24 January 2022) [57]. The steered molecular dynamics simulation (Str_mod) was chosen for one-nanosecond molecular dynamics simulation in an explicit water model.

## 4. Conclusions

In conclusion, all 10 selected AVPs named AVP1 to AVP10 from our proposed bioinformatic virtual screening workflow were quite diverse in length (7–33 amino acid residues). These top-ten AVP candidates contain hydrophilic amino acid residues and have a positive net charge. The molecular docking performances infer that all AVP candidates had significant hydrogen bonding with the SARS-CoV-2 main protease (PDB ID = 7C2Q) active site at Thr22–24, Glu154, and Thr178 in domain 2 with short bonding distances. AVP4 was the best candidate for SARS-CoV-2 Mpro inhibition with the highest affinity and binding energy among all selected AVPs. Further in vitro and in vivo studies must be conducted to authenticate the anti-COVID-19 potential of these AVP candidates. 

## Figures and Tables

**Table 1:** Predictive probability scores or SVM scores from antiviral peptide prediction tools.

Peptide ID	Secondary Structures	Sequences (from Protein/Cut by)	Length	AVPpred		Meta-iAVP	AMP Fun	ENNAVIA			
				M3*	M4*			A*	B*	C*	D*
AVP1	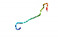	SWCRCSALNHMVGGIY (albumin/pepsin)	16	55.56	72.37	0.51	0.39	0.47	1.00	0.79	1.00
AVP2	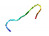	HQASSLLRGIKNY (globulin/pepsin)	13	54.82	24.41	0.98	0.49	0.97	0.74	0.57	0.23
AVP3	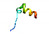	VVFSALLLIIVSVLAATATMADHHK (albumin/trypsin)	25	52.40	25.59	0.54	0.59	1.00	0.00	1.00	0.01
AVP4	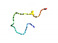	QQHSIVATPFWQPATFQLINNQVMQQQCCQQLR (prolamin/trypsin)	33	37.06	64.03	0.06	0.65	0.68	0.00	0.99	0.16
AVP5	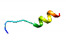	IIFVFALLAIVACNASAR (prolamin/pepsin/trypsin)	18	56.12	33.78	0.99	0.43	1.00	0.00	0.75	0.00
AVP6	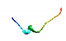	PILSLVQMSAVKVNLY (glutelin/pepsin)	16	46.62	5 5.77	0.32	0.53	0.10	0.00	0.80	0.32
AVP7	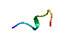	GGHGPHWPLPPF (globulin/pepsin)	12	42.04	25.90	1.00	0.51	0.74	0.91	0.00	0.08
AVP8	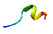	VIALPAGVAHWCY (glutelin/pepsin)	13	41.67	62.94	0.54	0.46	0.50	0.31	0.35	0.28
AVP9	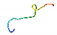	ATILLLLAAVLFAAAAAASGEDR (globulin/trypsin)	23	47.39	62.95	0.74	0.43	0.96	0.00	0.40	0.00
AVP10	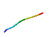	QQVGVVY (prolamin/chymotrypsin)	7	50.46	43.09	0.99	0.66	0.05	0.20	0.20	0.45

Note: For the AVPpred program; M3* = composition of the SVM-based models (Model 3), M4* = physiochemical property SVM-based models (Model 4). For the ENNAVIA program, the neural network models based on the antiviral vs. non-antiviral datasets (A*), antiviral vs. random datasets (B*), anti-coronavirus vs. non-antiviral datasets (C*), and anti-coronavirus vs. random datasets (D*).

**Table 2:** Calculated physicochemical property scores of the selected AVP candidates (AVP1 to AVP10) by ToxinPred.

Peptide ID	Hydrophobicity	Steric Hindrance	Sidebulk	Hydropathicity	Amphipathicity	Hydrophilicity	Net Hydrogen	Charge	pI	Mol wt
AVP1	−0.01	0.6	0.6	0.34	0.24	−0.71	0.69	1.5	8.38	1797.33
AVP2	−0.22	0.58	0.58	−0.62	0.68	−0.13	1.08	2.5	10.01	1486.89
AVP3	0.17	0.56	0.56	1.52	0.26	−0.73	0.36	1	7.26	2621.56
AVP4	−0.15	0.61	0.61	−0.45	0.5	−0.56	1	1.5	8.4	3916.01
AVP5	0.23	0.62	0.62	2.1	0.14	−0.94	0.39	1	8.6	1892.59
AVP6	0.09	0.62	0.62	1.05	0.31	−0.74	0.56	1	8.94	1775.43
AVP7	0.08	0.43	0.43	−0.69	0.24	−0.72	0.25	1	7.26	1298.65
AVP8	0.25	0.54	0.54	1.32	0.11	−1.18	0.23	0.5	7.06	1399.86
AVP9	0.15	0.58	0.58	1.45	0.16	−0.45	0.35	−1	4.38	2228.94
AVP10	0.06	0.69	0.69	0.56	0.36	−0.91	0.71	0	5.88	792.01

**Table 3:** List of hydrogen bonds observed from the molecular docking of ten peptides (AVP1 to AVP10) to the crystal structure of the SARS-CoV-2 main protease in the apo state (PDB ID: 7C2Q).

Peptides	Peptide Residues	Protease Residues	Distance (Å)	Peptides	Peptide Residues	Protease Residues	Distance (Å)
AVP1	Arg4	Gln180	2.485	AVP4	Arg33	Thr19	2.285
	Ser6	Thr178	2.124	(continue)	Arg33	Thr24	2.335
	Ala7	Glu154	1.971		Arg33	Gln61	2.041
	His10	Cys42	2.978		Arg33	Thr19	2.018
	Val12	Gly131	2.124	AVP5	Ile1	Ala181	2.404
AVP2	Ser4	Gln180	1.976		Asn14	Thr24	2.085
	Leu6	Glu154	1.912	AVP6	Pro1	Gln177	1.917
	Arg8	His151	2.036		Ile2	Glu154	2.013
	Arg8	His160	2.047		Val6	Asn111	2.428
	Ile10	Thr24	2.078		Gln7	Thr24	1.902
	Tyr13	His39	2.778		Ala10	Thr24	2.463
AVP3	Ala5	Thr178	2.410		Lys12	Gly21	2.137
	Leu6	Glu154	1.875		Lys12	Asn57	1.939
	Ser12	Asn111	2.330		Tyr16	Asn55	2.536
	Ala15	Thr24	2.045		Tyr16	Val69	1.995
	Thr19	Thr22	2.017	AVP7	Phe12	Gly131	2.526
AVP4	Ser4	Val286	2.047		Phe12	Cys133	1.924
	Thr8	Pro156	2.031		Phe12	His152	2.091
	Trp11	Ser274	2.279	AVP8	Val1	Asp209	1.898
	Gln12	Asp209	2.021		Val1	Thr157	1.975
	Pro13	Arg123	2.196		Cys12	Thr23	3.071
	Thr15	Ala182	2.471	AVP9	Ala1	Arp209	2.000
	Thr15	Gly183	1.938		Ala1	Thr157	1.861
	Asn20	Gln180	2.430		Thr2	Gly271	2.093
	Gln22	Gln180	2.057		Thr2	Arp185	1.972
	Gln22	Gln177	2.493		Val10	Glu154	1.894
	Gln22	Thr178	2.523		Ala13	Thr24	2.390
	Gln22	Asp175	2.064		Ser19	Asn111	2.178
	Val23	Glu154	1.935		Glu21	Thr24	2.242
	Gln25	Ser132	2.372		Arp22	Gln61	2.349
	Gln25	Glu154	2.035		Arg23	Gln17	2.163
	Gln26	Cys42	2.026		Arg23	Asn64	2.379
	Cys28	Glu154	3.828	AVP10	Gln1	Ala179	2.554
	Gln30	Thr24	1.993		Val5	Glu154	1.868
	Gln31	Thr22	2.426		Tyr7	Ser132	2.501
	Gln31	Thr23	2.093		Tyr7	Glu154	2.111

**Table 4:** Calculated binding affinity (ΔG), dissociation constant (Kd), and binding energy scores from the molecular docking of ten peptides (AVP1 to AVP10) to the SARS-CoV-2 main protease based on the PROGIDY and PIMA web servers. Molecular docking scores, H-bonds, electrostatic energy, and van der Waals energy (H-bond Ener., Elec. Ener., and VDW. Ener.) are presented in kJ/mol.

Protein-Peptide Complex	ΔG (kcal/mol)	Kd (M) at 25.0 °C	H-Bond Ener. (kJ/mol)	Elec. Ener. (kJ/mol)	VDW. Ener. (kJ/mol)	Molecular Docking Score (kJ/mol)
Mpro–AVP1	−11.2	5.8E−09	−23.62	4.48	−206.06	−225.20
Mpro–AVP2	−10.5	1.9E−08	−31.67	2.43	−196.55	−225.79
Mpro–AVP3	−11.0	9.3E−09	−27.37	0.00	−153.91	−181.28
Mpro–AVP4	−14.5	2.2E−11	−37.56	0.00	−325.48	−363.04
Mpro–AVP5	−9.9	5.7E−08	−3.19	0.00	−160.49	−163.68
Mpro–AVP6	−11.0	9.1E−09	−42.27	4.91	−187.82	−225.19
Mpro–AVP7	−8.2	9.3E−07	−23.68	0.00	−133.58	−157.26
Mpro–AVP8	−10.0	4.5E−08	−6.39	−13.14	−107.89	−127.42
Mpro–AVP9	−12.6	5.9E−10	−23.33	0.00	−219.64	−242.97
Mpro–AVP10	−9.4	1.3E−07	−25.32	0.00	−109.52	−134.84

**Table 5:** Predictive SVM and QM scores from ToxinPred.

Peptide ID	SVM Method				Quantitative Matrix (QM) Method			
	Model A*	Model B*	Model C*	Model D*	Model E*	Model F*	Model G*	Model H*
AVP1	−0.22	−0.22	−0.28	−0.28	19.70	9.06	0.33	−0.91
AVP2	−0.94	−0.94	−1.16	−1.16	−14.50	−12.95	−3.82	−2.13
AVP3	−1.40	−1.40	−1.77	−1.77	−27.60	−46.75	−4.69	−7.79
AVP4	−1.07	−1.07	−1.14	−1.14	−1.90	−17.13	−1.64	−1.91
AVP5	−0.68	−0.68	−0.68	−0.68	−8.50	−13.44	−2.30	−2.32
AVP6	−1.31	−1.31	−2.03	−2.03	−15.70	−23.15	−0.32	−1.35
AVP7	−0.75	−0.75	−1.30	−1.30	12.50	22.19	1.28	1.85
AVP8	−0.19	−0.19	−0.61	−0.61	7.70	9.28	−1.36	0.43
AVP9	−1.69	−1.69	−1.38	−1.38	−42.50	−52.14	−9.10	−10.11
AVP10	−1.45	−1.45	−1.18	−1.18	−16.00	−7.16	−0.91	−0.47

Note: Moldel A = SVM (Swiss-Prot) based, Model B = SVM (Swiss-Prot) + Motif based, Model C = SVM (TrEMBL) based, Model D = SVM (TrEMBL) + Motif based, Model E = Monopeptide (Swiss-Prot), Model F = Monopeptide (TrEMBL), Model G = Dipeptide (Swiss-Prot), and Model H = Dipeptide (TrEMBL).

## Supplementary Materials

The following are available online at https://www.mdpi.com/article/10.3390/antibiotics11101318/s1, Table S1: Total peptide sequences of the putative peptidome of rice bran (*Oryza sativa*) hydrolyzed by pepsin, trypsin, and chymotrypsin.
