# Supplementary material for: Virtual Screening for SARS-CoV-2 Main Protease Inhibitory Peptides from the Putative Hydrolyzed Peptidome of Rice Bran
Source: Antibiotics (Basel). 2022 Sep 27;11(10):1318. doi: 10.3390/antibiotics11101318 (PMC9598432; doi:10.3390/antibiotics11101318)
Supplement: Supplementary file 1 [file antibiotics-11-01318-s001.zip › antibiotics-1920339-supplementary.pdf]

Table S1. Total peptide sequences from the putative peptidome of rice bran (*Oryza sativa*) hydrolyzed by pepsin, trypsin, and chymotrypsin

ID	Sequences	length (aa)	original protein	digested by
seq1	AAAAAASGE	9	Globulin	Pepsin
seq2	AAAAAASGEDRRRETSL	17	Globulin	Chymotrypsin
seq3	AAASLPAFCNVDIPNGTGGVCYWLGYPR	28	Albumin	Trypsin
seq4	AASARE	6	Globulin	Pepsin
seq5	AASAREVDEL	10	Globulin	Chymotrypsin
seq6	AATATMADHHKDQVVY	16	Albumin	Chymotrypsin
seq7	AAVDDSW	7	Albumin	Chymotrypsin
seq8	ADTYNPR	7	Glutelin	Trypsin
seq9	AEHDIRVAVVNITAGSMNAPF	21	Globulin	Chymotrypsin
seq10	AGMNSVL	7	Globulin	Chymotrypsin
seq11	AGNKRNPQAY	10	Glutelin	Chymotrypsin
seq12	LLPPFHQASSLLR	13	Globulin	Trypsin
seq13	AHCICY	6	Globulin	Pepsin
seq14	AIRQGD	6	Globulin	Pepsin
seq15	AIRQGDVF	8	Globulin	Chymotrypsin
seq16	AIVACNASARF	11	Prolamin	Chymotrypsin
seq17	ALGVSSQVARQLQCQND	17	Glutelin	Pepsin
seq18	ALLAIVACNASARF	14	Prolamin	Pepsin
seq19	ALPNDVLANAYR	12	Glutelin	Trypsin
seq20	ALSQSY	6	Prolamin	Pepsin
seq21	ANPRSF	6	Globulin	Pepsin
seq22	ANRHGRL	7	Globulin	Chymotrypsin
seq23	ANRHGRLY	8	Globulin	Pepsin
seq24	APGGRNPE	8	Globulin	Pepsin
seq25	APGGRNPESF	10	Globulin	Chymotrypsin
seq26	AQRLKHNRGD	10	Glutelin	Pepsin
seq27	ASEEQVR	7	Globulin	Trypsin
seq28	ASLQE	5	Glutelin	Pepsin
seq29	ATILLLLAAVLFAAAAAASGEDR	23	Globulin	Trypsin
seq30	AVVKKAQREGCAY	13	Glutelin	Chymotrypsin
seq31	CCRQLAAVD	9	Albumin	Pepsin
seq32	CEQDR	5	Globulin	Trypsin
seq33	CNGSL	5	Glutelin	Chymotrypsin
seq34	CNVDIPNGTGGVCY	14	Albumin	Chymotrypsin
seq35	CQPGMGYPMYSLPR	14	Albumin	Trypsin
seq36	CRCSAL	6	Albumin	Chymotrypsin
seq37	CTLRVRQNID	10	Glutelin	Pepsin
seq38	CVQECK	6	Globulin	Trypsin
seq39	DEHQK	5	Glutelin	Trypsin
seq40	DFLLAGNK	8	Glutelin	Trypsin
seq41	DGEGEAEIVCPHL	13	Globulin	Chymotrypsin
seq42	DPQAKEL	7	Globulin	Chymotrypsin
seq43	DPRQRDF	7	Glutelin	Chymotrypsin
seq44	DQQQQQQER	9	Globulin	Trypsin
seq45	DQTQAQAQAL	10	Prolamin	Chymotrypsin
seq46	DQVVYSLGER	10	Albumin	Trypsin
seq47	DSTLQIVCFDVHANNNER	18	Globulin	Trypsin

ID	Sequences	length (aa)	original protein	digested by
seq48	DVHANNNERMY	11	Globulin	Chymotrypsin
seq49	DVSNEQF	7	Glutelin	Chymotrypsin
seq50	EADARSF	7	Globulin	Chymotrypsin
seq51	EANPRSF	7	Globulin	Chymotrypsin
seq52	EEAQR	5	Glutelin	Trypsin
seq53	EEEEEEEEQQK	11	Globulin	Trypsin
seq54	EGCAYIAFK	9	Glutelin	Trypsin
seq55	EHGGHDDDR	9	Globulin	Trypsin
seq56	ELAFAASAR	9	Globulin	Trypsin
seq57	ELGATDVGHPMAEVFPGCR	19	Albumin	Trypsin
seq58	EPIRSVRSQAGTTEF	15	Glutelin	Chymotrypsin
seq59	EQRPRF	6	Globulin	Chymotrypsin
seq60	ERAAASL	7	Albumin	Chymotrypsin
seq61	ETIRARL	7	Globulin	Chymotrypsin
seq62	ETSLR	5	Globulin	Trypsin
seq63	EVDELLNAQQESAFLAGPEK	20	Globulin	Trypsin
seq64	EVEER	5	Glutelin	Trypsin
seq65	FDALSQSYR	9	Prolamin	Trypsin
seq66	GATDVGHPMAEVF	13	Albumin	Chymotrypsin
seq67	GDEAVETLLR	10	Globulin	Trypsin
seq68	GDEFGAFTPIQYK	13	Glutelin	Trypsin
seq69	GDLER	5	Albumin	Trypsin
seq70	GEESEDEDR	9	Globulin	Trypsin
seq71	GEGSSEEEDEGR	12	Globulin	Trypsin
seq72	GEIVR	5	Glutelin	Trypsin
seq73	GERCQPGMGY	10	Albumin	Chymotrypsin
seq74	GESSRGPF	8	Globulin	Chymotrypsin
seq75	GGESEER	7	Globulin	Trypsin
seq76	GGHGPHWPLPPF	12	Globulin	Pepsin
seq77	TNPNSMVSHIAGK	13	Glutelin	Trypsin
seq78	GKQDKGVIIRASEEQVREL	19	Globulin	Chymotrypsin
seq79	GLLLPHYTNGASLVYIIQGR	20	Glutelin	Trypsin
seq80	GPFNILEQR	9	Globulin	Trypsin
seq81	GQEEEEEEQVGQGYETIR	18	Globulin	Trypsin
seq82	GQLLIIPQHYAVVK	14	Glutelin	Trypsin
seq83	GQSTSQW	7	Glutelin	Chymotrypsin
seq84	GRRSF	5	Globulin	Chymotrypsin
seq85	GSGCSNGL	8	Glutelin	Chymotrypsin
seq86	GSGCSNGLD	9	Glutelin	Pepsin
seq87	GTVFVVPSGHPIVVTSSR	18	Globulin	Trypsin
seq88	GVIIR	5	Globulin	Trypsin
seq89	GVSSQVARQL	10	Glutelin	Chymotrypsin
seq90	GVVAIIE	7	Globulin	Pepsin
seq91	HASEGGHGPHWPLPPFGESSR	21	Globulin	Trypsin
seq92	HGGHD	5	Globulin	Pepsin
seq93	HGLSLLQPY	9	Glutelin	Pepsin
seq94	HQASSL	6	Globulin	Chymotrypsin
seq96	HTISVPGQIQF	11	Globulin	Chymotrypsin
seq97	IAPRSIPTVGGVW	13	Prolamin	Chymotrypsin

ID	Sequences	length (aa)	original protein	digested by
seq98	IAPRSIPTVGGVWY	14	Prolamin	Pepsin
seq99	IIFVFALLAIVACNASAR	18	Prolamin	Trypsin
seq100	IIPQHY	6	Glutelin	Chymotrypsin
seq101	IIQGRGITGPTF	12	Glutelin	Chymotrypsin
seq102	IIVSVL	6	Albumin	Chymotrypsin
seq103	ILHTISVPGQIQFFFAPGGR	20	Globulin	Trypsin
seq104	INNQVMQQQCCQQL	14	Prolamin	Chymotrypsin
seq105	IPNGTGGVCY	10	Albumin	Pepsin
seq106	IRVAVVNITAGSMNAPF	17	Globulin	Pepsin
seq107	ISEEK	5	Globulin	Trypsin
seq108	IVCPHLSRGGRGGE	14	Globulin	Pepsin
seq109	IVRVE	5	Glutelin	Pepsin
seq110	KDEHQKIHRF	10	Glutelin	Chymotrypsin
seq111	KGVIIRASE	9	Globulin	Pepsin
seq112	KHNRGDEF	8	Glutelin	Chymotrypsin
seq113	KISEEKL	7	Globulin	Chymotrypsin
seq114	KSGRRGE	7	Globulin	Pepsin
seq115	KTNPNSMVSHIAGKSSIF	18	Glutelin	Chymotrypsin
seq116	LAGPE	5	Globulin	Pepsin
seq117	LANTD	5	Globulin	Pepsin
seq118	LDPQAK	6	Globulin	Trypsin
seq119	LGATD	5	Albumin	Pepsin
seq120	LIVTK	5	Globulin	Trypsin
seq121	LLAGNKRNPQAY	12	Glutelin	Pepsin
seq122	LLCNGSLAQQLLGQSTSQWQSSRRGSPRE	29	Glutelin	Pepsin
seq123	LLNAQQE	7	Globulin	Pepsin
seq124	LNNGANQLD	9	Glutelin	Pepsin
seq125	LQAFEPIR	8	Glutelin	Trypsin
seq126	LRRGQLLIIPQHY	13	Glutelin	Pepsin
seq127	LRRHASE	7	Globulin	Pepsin
seq128	LYEADAR	7	Globulin	Trypsin
seq129	MAAAAV	6	Globulin	Trypsin
seq130	MASINR	6	Glutelin	Trypsin
seq131	MASINRPIVF	10	Glutelin	Chymotrypsin
seq132	MASNK	5	Albumin	Trypsin
seq133	MASNKVVF	8	Albumin	Chymotrypsin
seq134	MATRARATIL	10	Globulin	Chymotrypsin
seq135	MATRARATILLLLLAAVLF	18	Globulin	Pepsin
seq136	MYLAGMNSVLK	11	Globulin	Trypsin
seq137	NAAESS	6	Glutelin	Chymotrypsin
seq138	NAQQESAF	8	Globulin	Chymotrypsin
seq139	NDGEVPVVAIY	11	Glutelin	Chymotrypsin
seq140	NHMVGGIY	8	Albumin	Chymotrypsin
seq141	NINAHSVVY	9	Glutelin	Chymotrypsin
seq142	NNGANQL	7	Glutelin	Chymotrypsin
seq143	NPESFLSSFSK	11	Globulin	Trypsin
seq144	NPNRAD	6	Glutelin	Pepsin
seq145	NPQAYR	6	Glutelin	Trypsin
seq146	NPRAGRVTNLNTQNF	15	Glutelin	Pepsin

ID	Sequences	length (aa)	original protein	digested by
seq147	NTQNF	5	Glutelin	Chymotrypsin
seq148	NTRSVKVAY	9	Globulin	Chymotrypsin
seq149	PAGVAHW	7	Glutelin	Chymotrypsin
seq150	PGCPE	5	Glutelin	Pepsin
seq151	PGCPESY	7	Glutelin	Chymotrypsin
seq152	PGCRRGD	7	Albumin	Pepsin
seq153	PGCRRGDL	8	Albumin	Chymotrypsin
seq154	PILSLVQMSAVKVNLY	16	Glutelin	Pepsin
seq155	PIRSVRSQAGTTE	13	Glutelin	Pepsin
seq156	SKGVQRAAF	9	Globulin	Chymotrypsin
seq157	PNDVL	5	Glutelin	Chymotrypsin
seq158	PPYER	5	Globulin	Trypsin
seq159	PQAKE	5	Globulin	Pepsin
seq160	PRCRAVVKRQCVGHGAPGGAVDEQL	25	Albumin	Chymotrypsin
seq161	PRGLLLPHY	9	Glutelin	Pepsin
seq162	PRQRD	5	Glutelin	Pepsin
seq163	PRTPRTGH	8	Albumin	Chymotrypsin
seq164	PSICGIY	7	Prolamin	Chymotrypsin
seq165	PYVFGR	6	Globulin	Trypsin
seq166	QAISSVQAIVQQL	13	Prolamin	Chymotrypsin
seq167	QAISSVQAIVQQLQLQQVGVVY	22	Prolamin	Pepsin
seq168	QCQNDQRGEIVRVEHGL	17	Glutelin	Chymotrypsin
seq169	QCTGVSVVRRVIE	13	Glutelin	Pepsin
seq170	QCTGVSVVRRVIEPRGL	17	Glutelin	Chymotrypsin
seq171	QCVGHGAPGGAVDEQLR	17	Albumin	Trypsin
seq172	QDCCR	5	Albumin	Trypsin
seq173	QEGQY	5	Glutelin	Chymotrypsin
seq174	QEQEQGQVQSRERY	14	Glutelin	Chymotrypsin
seq175	QGDVFVAPAGTINYLANTDGR	21	Globulin	Trypsin
seq176	TSLRRCLQRCE	11	Globulin	Pepsin
seq177	QGQVQSRE	8	Glutelin	Pepsin
seq178	QGSVRLLPPF	10	Globulin	Pepsin
seq179	QIVCF	5	Globulin	Chymotrypsin
seq180	QLAAVDDSWCR	11	Albumin	Trypsin
seq181	QLINNQVMQQQCCQQLRLVAQQSHY	25	Prolamin	Pepsin
seq182	QLQCQNDQR	9	Glutelin	Trypsin
seq183	QLQSHLLLQQQVLSPCSE	18	Prolamin	Pepsin
seq184	QLRQD	5	Albumin	Pepsin
seq185	QNALLSPF	8	Glutelin	Pepsin
seq186	QNIDNPNR	8	Glutelin	Trypsin
seq187	QPATF	5	Prolamin	Chymotrypsin
seq188	ITQGRARVQVVNNNGKTVF	19	Glutelin	Chymotrypsin
seq189	QQKGQE	6	Globulin	Pepsin
seq190	QQQQQQE	7	Globulin	Pepsin
seq191	QQQVL	5	Prolamin	Chymotrypsin
seq192	QQSGQAQL	8	Glutelin	Chymotrypsin
seq193	QQSGQAQLTE	10	Glutelin	Pepsin
seq194	QQSQY	5	Glutelin	Chymotrypsin
seq195	QQVGVVY	7	Prolamin	Chymotrypsin

ID	Sequences	length (aa)	original protein	digested by
seq196	QRCEQDRPPY	10	Globulin	Chymotrypsin
seq197	QRPRF	5	Globulin	Pepsin
seq198	QSSRRGSPRECRF	13	Glutelin	Chymotrypsin
seq199	QTQAQAQALLALNLPSICGIY	21	Prolamin	Pepsin
seq200	QVGQGY	6	Globulin	Pepsin
seq201	QYQLQSHLLLQQQVLSPCSEFVR	23	Prolamin	Trypsin
seq202	RAAASLPAF	9	Albumin	Pepsin
seq203	RALPND	6	Glutelin	Pepsin
seq204	RARCVQE	7	Globulin	Pepsin
seq205	RCQPGMGY	8	Albumin	Pepsin
seq206	REEEEEEEEQQKGQEEEEEEQVGQGY	26	Globulin	Chymotrypsin
seq207	RGKGKWRE	8	Globulin	Pepsin
seq208	RGSRRRPY	8	Globulin	Pepsin
seq209	RISRE	5	Glutelin	Pepsin
seq210	RISREEAQRL	10	Glutelin	Chymotrypsin
seq211	RLQAF	5	Glutelin	Pepsin
seq212	RMAAAAV	7	Globulin	Chymotrypsin
seq213	RQDCCRQL	8	Albumin	Chymotrypsin
seq214	RQGDVIAL	8	Glutelin	Chymotrypsin
seq215	RQVVRSD	7	Globulin	Pepsin
seq216	RQVVRSDQGSVRL	13	Globulin	Chymotrypsin
seq217	RREVEERSQNIF	12	Glutelin	Chymotrypsin
seq218	RRGQL	5	Glutelin	Chymotrypsin
seq219	RRHASEGGHGPHW	13	Globulin	Chymotrypsin
seq220	RRRRRSHRGRGD	12	Globulin	Pepsin
seq221	RSQNIF	6	Glutelin	Pepsin
seq222	RVAVL	5	Globulin	Chymotrypsin
seq223	RVAVLE	6	Globulin	Pepsin
seq224	RVRQNIDNPNRADTY	15	Glutelin	Chymotrypsin
seq225	SALLLIIVSVLAATATMAD	19	Albumin	Pepsin
seq226	SDQGSVR	7	Globulin	Trypsin
seq227	SFHDLAEHDIR	11	Globulin	Trypsin
seq228	AVVKKAQRE	9	Glutelin	Pepsin
seq229	SIPTVGGVWY	10	Prolamin	Trypsin
seq230	SLPRCRAVVKRQCVGHGAPGGAVD	24	Albumin	Pepsin
seq231	SPCSEF	6	Prolamin	Chymotrypsin
seq232	SQAGTTEFFDVSNEQFQCTGVSVVR	25	Glutelin	Trypsin
seq233	SQNIFSGFSTELLSEALGVSSQVAR	25	Glutelin	Trypsin
seq234	SQSQSQKF	8	Glutelin	Pepsin
seq235	SRGGRGGESEERRRERGKGKW	21	Globulin	Chymotrypsin
seq236	SRGTVF	6	Globulin	Chymotrypsin
seq237	SSRGPF	6	Globulin	Pepsin
seq238	STLQIVCF	8	Globulin	Pepsin
seq239	SWCRCSALNHMVGGIY	16	Albumin	Pepsin
seq240	SYQDVYNAAESS	12	Glutelin	Trypsin
seq241	TESQSQSQKF	10	Glutelin	Chymotrypsin
seq242	TIRARLSRGTVF	12	Globulin	Pepsin
seq243	TLLRMAAAAV	10	Globulin	Pepsin
seq244	TNGASL	6	Glutelin	Chymotrypsin

ID	Sequences	length (aa)	original protein	digested by
seq245	TNGASLVY	8	Glutelin	Pepsin
seq246	TPIQY	5	Glutelin	Chymotrypsin
seq247	TVCLF	5	Glutelin	Pepsin
seq248	TVFNGELR	8	Glutelin	Trypsin
seq249	VAPAGTINY	9	Globulin	Chymotrypsin
seq250	VAQGE	5	Globulin	Pepsin
seq251	VAQGEGVVAIIENGEW	16	Globulin	Chymotrypsin
seq252	VAQQSHY	7	Prolamin	Chymotrypsin
seq253	VAVLEANPR	9	Globulin	Trypsin
seq254	VAVVNITAGSMNAPFYNTR	19	Globulin	Trypsin
seq255	VAYVLDGEGEAEIVCPHLSR	20	Globulin	Trypsin
seq256	VEHGLSLLQPYASLQEQEQGQVQSR	25	Glutelin	Trypsin
seq257	VGHPMAE	7	Albumin	Pepsin
seq258	VHANNNE	7	Globulin	Pepsin
seq259	VIALPAGVAHWCY	13	Glutelin	Pepsin
seq260	VIEPR	5	Glutelin	Trypsin
seq261	VLANAY	6	Glutelin	Pepsin
seq262	VMPTHTD	7	Globulin	Pepsin
seq263	VMPTHTDAHCICY	13	Globulin	Chymotrypsin
seq264	VNLYQNALLSPFWNINAHSVVYITQGR	27	Glutelin	Trypsin
seq265	VPVVAIY	7	Glutelin	Pepsin
seq266	VQMSAVKVNL	10	Glutelin	Chymotrypsin
seq267	VQVVNNNGK	9	Glutelin	Trypsin
seq268	VRQQHSIVATPF	12	Prolamin	Chymotrypsin
seq269	VTNLNTQNFPILSLVQMSAVK	21	Glutelin	Trypsin
seq270	VVFSALLLIIVSVLAATATMADHHK	25	Albumin	Trypsin
seq271	VVPSGHPIVVTSSRD	15	Globulin	Pepsin
seq272	VVPSGHPIVVTSSRDSTL	18	Globulin	Chymotrypsin
seq273	WNINAHSVVY	10	Glutelin	Pepsin
seq274	WQPATF	6	Prolamin	Pepsin
seq275	YQEGQYQQSQYGSGCSNGLDETFCTLR	27	Glutelin	Trypsin
seq276	ANTDGRRKL	9	Globulin	Chymotrypsin
seq277	NPRAGRVTNL	10	Glutelin	Chymotrypsin
seq278	CSALNHMVGGIYR	13	Albumin	Trypsin
seq279	GRRKLIVTKILHTISVPGQIQF	22	Globulin	Pepsin
seq280	LAGMNSVLKKLD	12	Globulin	Pepsin
seq281	KLLGKQD	7	Globulin	Pepsin
seq282	MKIIF	5	Prolamin	Chymotrypsin
seq283	SSIFR	5	Glutelin	Trypsin
seq284	RGIKNY	6	Globulin	Chymotrypsin
seq285	IVTKIL	6	Globulin	Chymotrypsin
seq286	HQKIHRF	7	Glutelin	Pepsin
seq287	PIVFFTVCLFLLCNGSLAQQLLGQSTSQWQ SSR	33	Glutelin	Trypsin
seq288	QQHSIVATPFWQPATFQLINNQVMQQQC CQQLR	33	Prolamin	Trypsin
seq289	AGPEKSGRRGEESEDEDRRRRRSHRGRGD EAVETl	35	Globulin	Chymotrypsin
seq290	GITGPTFPGCPESYQQQFQQSGQAQLTESQ SQSQK	35	Glutelin	Trypsin

ID	Sequences	length (aa)	original protein	digested by
seq291	SFVMPTHTDAHCICYVAQGEGVVAIIENG EWSYAIR	36	Globulin	Trypsin
seq292	QGDVIALPAGVAHWCYNDGEVPVVAIYV TDLNNGANQLDPR	41	Glutelin	Trypsin
